# Favorable early outcomes of medial unicompartimental knee arthroplasty in active patients presenting a degenerative medial meniscus root tear with meniscal extrusion and mild radiographic osteoarthritis

**DOI:** 10.1007/s00402-024-05233-5

**Published:** 2024-03-14

**Authors:** Matteo Innocenti, Filippo Leggieri, Kim Huber, Bernard Christen, Tilman Calliess

**Affiliations:** 1 Department of Orthopaedic Surgery, University of Florence, Florence, Italy; 2 Articon Spezialpraxis Für Gelenkchirurgie, Berne, Switzerland

**Keywords:** SONK, Root tear, Meniscal extrusion, UKA, Mild OA, Early OA

## Abstract

**Introduction:**

There is only limited literature available evaluating the preferable treatment for active mid-age or elderly patients presenting with a degenerative medial meniscus root tear (d-MMRT) with medial meniscal extrusion (MME) and early-phase radiographic osteoarthritis (OA), failing to provide solid evidence.

The aim of this study was to evaluate early outcomes of medial unicompartimental arthroplasty (mUKA) in active patients presenting a d-MMRT with meniscal extrusion and mild radiographic OA of the knee. To prove this claim we hypothesized that (1) patients with a d-MMRT with initial grade 1–3 KL OA of the medial compartment of the knee present the same pre-operative symptoms as patients with an end-stage grade 4 K-L OA, and that (2) those patients with d-MMRT and low-grade OA achieve the same early clinical and functional outcomes when treated with mUKA compared to patients with end-stage medial OA.

**Methods:**

We reviewed the prospectively collected data of 185 patients undergoing robotic-assisted image-based mUKA from January 2021 to July 2022 at a single Institution. We identified two different cohorts of non-consecutive patients: a group of 24 patients undergoing mUKA surgery following d-MMRT combined with K-L grades 1–3 OA (group 1), and a group of 161 patients who underwent mUKA but presenting with an end-stage bone-on-bone K-L grade 4 OA (group 2). Preoperative and postoperative clinical assessments at one-year follow-up included the Oxford Knee Score (OKS), 5-level EQ-5D version (EQ-5D-5L score), and a standard weight-bearing X-ray protocol. The paired t-test was used to compare clinical outcomes and radiologic values of the two cohorts and in-between the two cohorts. Statistical significance was set at *p* < 0.05.

**Results:**

The mean follow-up for group 1 was 16.4 ± 2.5 months, and the mean age at the time of operation was 63 ± 8.6 years with a mean time from diagnosis to intervention of 53 ± 47.5 days. Preoperative impairment was greater in group 1 compared to group 2, but with no significant difference. Postoperatively, both groups showed *excellent* mean outcomes at 1-year follow-up, with no significant difference. The mean preoperative HKA, mPTA, and LDFA were 176.3 ± 3.1, 88.1 ± 2.3, and 86.6 ± 1.7 respectively. The mean postoperative HKA, coronal femoral component angle, and coronal tibial component angle were 179.1 ± 2.6, 87.2 ± 2.3, and 87.2 ± 3.3. No difference was found between preoperative age, BMI, between the two cohorts.

**Conclusions:**

Favorable early clinical outcomes were obtained after mUKA in active mid-age and elderly patients presenting with degenerative medial meniscus root tear and mild isolated medial OA. Patients with mild no bone-on-bone OA but with degenerative medial meniscus root tear and medial meniscal extrusion presented the same or worse pre-operative symptoms as patients with end-stage medial OA and benefit the same from mUKA.

## Introduction

Medial meniscal extrusion (MME) refers to a radial medial meniscal body displacement off the peripheral tibial edge by more than 3 mm reducing its tibiofemoral contact area. Biomechanical studies have demonstrated that the medial meniscus transmits 50% of the joint load in the medial compartment and that at least 50% of the compressive load of the knee joint is transmitted through the meniscus in extension [1, 2]. As a consequence of a MME, the reduced tibiofemoral contact area increases mean and peak weight-bearing forces resulting in a diverged knee kinematic and ultimately leading to an increased risk of osteophytosis, chondral lesions, loss of cartilage, and eventually osteoarthritis (OA) [3–9].

MME frequently occurs in conjunction with radial medial meniscus tears (MMT), with an incidence of 46.7%, particularly in medial meniscus root tears (MMRT), up to 83.3% of cases [4].

Different viable treatment options are available for MMRT including non-operative treatment, partial meniscectomy, and MMRT repair [10]. Pullout repair remains the gold standard in the treatment of MMRT, conversely, non-operative or resecting treatment may lead to the degenerative changes already previously discussed [11–14].

Although MMRT repair results have been promising, indications for surgery are limited to young and active patients with an otherwise healthy knee [15]. In any case, the surgical repair of a MMRT remains associated with a long recovery time from surgery. Furthermore, a systematic review showing midterm outcomes of MMRT repair concluded that 49% of the included population had radiographic progression of at least 1 grade in the Kellgren-Lawrence scale at a mean follow-up of 4 years [16].

Limited mid-term evidence evaluating non-active young adults with meniscal extrusion and early phase radiographic OA reports good clinical outcomes after MMRT repair but fails to provide strong evidence in such population [14]. Based on the long recovery time and the increased risk of treatment failures in elderly patients with degenerative MMRT (d-MMRT), the ideal surgical solution for this collective remains under debate [11]. Possibly, medial unicompartimental arthroplasty (mUKA) with a predictable outcome and faster recovery to full-weight-bearing represents an interesting treatment option. However, performing a mUKA in a patient with no bone-on-bone grade 4 K-L OA has usually been considered out of the classical and recently expanded indications for performing a mUKA since it could be associated with poor outcomes as has already been widely demonstrated for total knee replacements [17–20].

The aim of this study was to evaluate early clinical outcomes of mUKA in active mid-age or elderly patients presenting a degenerative MMRT (d-MMRT) with meniscal extrusion and mild radiographic OA of the knee. The main two hypotheses were that (1) patients with a d-MMRT with initial grade 1–3 KL OA of the medial compartment of the knee present the same pre-operative symptoms and functional impairment as patients with an end-stage grade 4 K-L OA, and that (2) those patients with d-MMRT and low-grade OA achieve the same early clinical and functional outcomes when treated with a mUKA compared to patients with end-stage medial OA.

## Materials and methods

### Study design and population

We reviewed the prospectively collected data of 185 patients (185 knees) undergoing robotic-assisted image-based mUKA from January 2021 to July 2022 at a single Institution. From the database, we identified two different cohorts of non-consecutive patients: a group of 24 patients undergoing mUKA surgery following d-MMRT combined with K-L grades 1–3 OA and no bone-on-bone at the weight-bearing X-rays (group 1, cases) and a group of 161 patients who underwent the same surgical intervention but presenting with an end-stage bone-on-bone K-L grade 4 OA (group 2, controls).

Patients with at least 1-year follow-up were eligible for the recruitment. Patients with missing data, without informed consent, and with less than 1 year of follow-up were excluded. In group 1, a patient had a follow-up period of 330 days. To prevent potential bias resulting from missing values and considering the minimal difference between 330 days and 1 year, we intentionally included this patient in the final cohort.

In group 1, a root tear was considered to be present if the MRI demonstrated either a complete degenerative meniscus posterior root avulsion or a complete degenerative radial tear at the posterior horn of the medial meniscus with clinical symptoms that correlated to the MRI findings.

All patients in group 1 participated in a minimum three-month period of conservative treatment, which included, for example, knee unloader braces, physiotherapy, and analgesic or anti-inflammatory drugs and/or hyaluronic acid/corticosteroid injections. Following this initial treatment phase, and considering the outcomes achieved, mUKA surgery was subsequently taken into consideration.

The study has been developed following the ethical standards proposed by the Declaration of Helsinki and its later amendments. All patients had provided informed consent to the treatment protocol, the operation protocol, and the rehabilitation and follow-up plan. The database was fully anonymized, and all patients gave their informed consent to data collection, analysis, and their anonymous use for scientific purposes and publications.

### Surgical technique

All surgical procedures were performed with the patient in a supine position and the tourniquet was routinely inflated prior to the skin incision. Intravenous antibiotic prophylaxis was administered following the hospital protocol. mUKAs were performed by two senior experienced knee surgeons with high workflow in robotic-assisted knee arthroplasties with the MAKO® robotic assistance (Stryker®, Mahwah, USA). No diagnostic arthroscopies were performed prior to the mUKAs skin incision. A midline incision was made, and the knee joint was exposed using a mini medial-parapatellar arthrotomy as the preferred approach for every case. The cruciate ligaments were examined to confirm suitability for mUKA. The same fixed-bearing metal-backed cemented unicompartmental knee prosthesis was implanted (RESTORIS MCK partial knee, Stryker, Mahwah, USA) with the assistance of the MAKO Robotic system. A classical workflow for a MAKO robotic-assisted surgery was used to position the arrays and to register and match the bone anatomy.

Kinematic data about Range Of Motion (ROM) and soft tissue laxity were recorded. After that, all osteophytes were resected, and the ROM and the kinematic data of the knee were acquired by applying varus-valgus stress to reduce the arthritic deformity. The aim was to restore coronal alignment with a maximum of 4.5° varus position of the tibial component and to restore the normal kinematics in extension and flexion with the desired laxity pattern between femoral and tibial components throughout the full ROM. Once bone resection was completed, trial implants were tested with robotic assistance feedback, and final implants were then cemented. In three cases a patellofemoral joint replacement was performed in addition to medial UKA due to a simultaneous OA deterioration of the third compartment.

All patients followed the same post-operative physical therapy protocol with free active/passive knee mobilization, quad strength exercises, and full weight-bearing as tolerated from the first postoperative day. All patients were discharged home from the hospital.

### Clinical and radiological assessment

Preoperative and postoperative clinical assessment included Oxford Knee Score (OKS) [21], 5-level EQ-5D version (EQ-5D-5L score) [22]. At 1 year of follow-up, the Swiss Orthopaedics Minimal Dataset (SOMD) questionnaire was also administered. The SOMD questionnaire is a standardized, generic, and patient-reported outcome questionnaire, comprising ten items such as the location of the disease, the level of pain within the past four weeks, the limitations at work/leisure/sleep/autonomy, the subjective value of a body part, the employment status, the work disability, and household support [23].

A classical radiographic examination with the antero-posterior (AP) standing and latero-lateral (LL) view in 90° flexion of the knee and the full-leg weight bearing AP view were performed to evaluate preoperative mMPTA and mLDFA pre- and postoperative HKA, and postoperative coronal femoral component angle (CFA), and the coronal tibial component angle (CTA) [24]. The preoperative OA assessment was performed according to the Kellgren–Lawrence classification [25].

The preoperative imaging evaluation also included an MRI to better evaluate the meniscal state of the affected knee in case the patients did not show a K-L grade 4 bone-on-bone OA at the weight-bearing X-rays.

Any mispositioning of the implanted prosthesis and any zone of radiolucency in the femoral or tibial component, endosteal cavitation, fractures or bone penetration, wearing, and loosening were collected. Any complication either clinical or radiological was evaluated and recorded.

### Statistical analysis

Statistical analyses were performed using SPSS software (IBM Corp. Released 2017. IBM SPSS Statistics for Windows, Version 25.0. Armonk, NY: IBM Corp.). Data are expressed as mean ± standard deviation (range, max–min) unless otherwise indicated. Statistical significance was set at *p* < 0.05. The paired t-test was used to compare preoperative clinical outcomes and radiologic values of the two cohorts and postoperative clinical outcomes and radiographic values of the two cohorts and preoperative and postoperative clinical outcomes and radiological values in-between the two cohorts. Chi-square analysis and likelihood ratio test were conducted to compare the responses on the Swiss Orthopaedics Minimal Dataset Satisfaction Questionnaire between group 1 and group 2.

## Results

Overall, 185 patients (185 knees) were identified for the analysis. The case cohort included 24 patients in group 1 and 161 control patients in group 2. Patient demographics are shown as frequencies of both cohorts in Table 1.

**Table 1:** Distribution of the population included in both cohorts. Values are shown as mean ± standard deviation (range). BMI = Body Mass Index

	Group 1	Group 2
Patients (n)	24	161
Male/Female(%)	33/66	43/57
Age at surgery (y)	63 ± 8.6 (46–83)	68 ± 8.2 (46–86)
Side Left/Right (%)	56/54	46/54
BMI (weight/height^2^)	30 ± 7.7 (19–52)	28 ± 4.9 (18–52)

In group 1 the mean follow-up period was 16.4 ± 2.5 months (12–23.8 months). The mean age of patients at the time of operation was 63 ± 8.6 years (46–83 years). The mean time from the day when the surgery was scheduled to the actual intervention date was 53 ± 47.5 days (12–225 days). Spontaneous osteonecrosis of the knee (SONK) was observed in 6 knees on preoperative radiologic examination. The preoperative Kellgren-Lawrence classification distribution is shown in Table 2. The preoperative clinical situation was associated with lower OKS and EQ-5D scores in group 1 compared to group 2, without reaching statistical significance (OKS 22.9 ± 7.8 vs. 27 ± 5.5, *p* = 0.085, EQ-5D 0.5 ± 0.2 vs. 0.6 ± 0.1, *p* = 0.067, Table 3 and 5). Clinical outcomes presenting a statistically significant improvement postoperatively in both cohorts are shown in Table 3. Both groups achieved *excellent* clinical outcomes, again without any differences between the cohorts (OKS 40.9 ± 5.5 vs. 41.1 ± 5, *p* = 0.482, EQ-5D 0.9 ± 0.1 vs. 0.8 ± 0.1, *p* = 0.572). At the final follow-up, knee extension among group 1 was − 0.75° ± 1.5° (0–3°) and knee flexion was 133° ± 7.6° (115–140°). Group 1 radiographic outcomes and the Swiss Orthopaedics Minimal Dataset (SOMD) questionnaire are presented respectively in Table 4 and 5.

**Table 2:** Kellgren-Lawrence classification among the patients of group 1 (cases) is shown as frequencies and percentages

Kellgren-Lawrence classification		
	Frequency (n)	Percentage (%)
Grade 1	3	12.5
Grade 2	14	58.3
Grade 3	7	29.2
Grade 4	0	0
Total	24	100

**Table 3:** Preoperative and postoperative PROMs in the cohorts. Means values are shown as mean ± standard deviation. C.I. 95% is shown as superior-inferior limits. C.I. 95% = Confidence Interval 95% dF = degrees of Freedom

Clinical scores						
Group 1	Preoperative	Postoperative	P values	T-test mean	C.I. 95%	dF
OKS	22.9 ± 7.8	40.9 ± 5.5	0.0	18.0 ± 7.4	21–14	22
EQ-5D-5L	0.5 ± 0.2	0.9 ± 0.1	0.0	0.3 ± 0.3	0.5–0.2	23
Group 2						
OKS	27.0 ± 5.5	41.1 ± 5.0	0.0	16.2 ± 7.9	17–14	145
EQ-5D-5L	0.6 ± 0.1	0.8 ± 0.1	0.0	0.3 ± 0.2	0.3–0.1	128

**Table 4:** Group 1 radiographic scores values are shown as mean ± standard deviation. C.I. 95% = Confidence Interval 95%, dF = degrees of Freedom

Radiografic measurement						
	Preoperative	Postoperative	T-test mean	P values	C.I. 95%	dF
HKA (°)	176.3 ± 3.1	179.1 ± 2.8	2.8 ± 2.6	0.00	3.9/1.7	23
mPTA(°)	88.13 ± 2.3	87.2 ± 2.3	0.9 ± 3.2	0.21	0.1/1.16	23
mLDFA	86.6 ± 1.7	87.2 ± 3.3	− 0.7 ± 1.8	0.332	− 2.0/0.7	23

**Table 5:** Swiss Orthopaedics Minimal Dataset Satisfaction Questionnaire for group 1

Satisfaction Questionnaire		Frequency (n)	Percentage (%)
How satisfied are you right now with the strength of your knee pain when sitting?	• Very satisfied • Satisfied • Neither satisfied nor dissatisfied • Dissatisfied	19 5 0 0	79% 21% 0 0
How satisfied are you right now with the strength of your knee pain when you are in bed?	• Very satisfied • Satisfied • Neither satisfied nor dissatisfied • Dissatisfied	16 8 0 0	66% 33% 0 0
How satisfied are you right now with the strength of your knee pain when you get out of the bed?	• Very satisfied • Satisfied • Neither satisfied nor dissatisfied • Dissatisfied	12 10 1 1	50% 41.6% 4.2% 4.2%
How satisfied are you currently with the strength of your knee pain when doing light housework?	• Very satisfied • Satisfied • Neither satisfied nor dissatisfied • Dissatisfied	16 8 0 0	66% 33% 0 0
How satisfied are you at the moment with the strength of your knee pain when doing recreational activities?	• Very satisfied • Satisfied • Neither satisfied nor dissatisfied • Dissatisfied	10 10 4 0	41.7% 41.7% 16.6% 0%
My expectations regarding the feasibility to do normal everyday activities were…	• Way too low—I am doing a lot better than expected • Too low—I am doing better than expected • Just right—My expectations are met • A bit too high—I am doing a little worse than expected	2 0 18 4	8.3% 0% 75% 16.7%
My expectations regarding the feasibility to do normal everyday activities were…	• Way too low—I am doing a lot better than expected • Too low—I am doing better than expected • Just right—My expectations are met • A bit too high—I am doing a little worse than expected	3 2 16 3	12.5% 8.3% 66.7% 12.5%
My expectations regarding the feasibility to do leisure and sports activities were…	• Way too low—I am doing a lot better than expected • Too low—I am doing better than expected • Just right—My expectations are met • A bit too high—I am doing a little worse than expected	3 2 14 5	12.5% 8.3% 58.3% 20.8%

No difference was found between preoperative BMI, OKS, and EQ-5D-5L scores and postoperative OKS and EQ-5D-5L scores between the two cohorts as shown in Table 6, but a significant statistical difference was observed in the ages of the two cohorts.

**Table 6:** Matched pair t-test for BMI, age, preoperative and postoperative PROMs between cohort are shown below. Mean values are expressed as mean ± standard deviation

	Mean	C.I. 95%		t	p-value
BMI	2.9 ± 8.9	− 6.79	0.8	− 1.6	0.124
Age	− 4.9 ± 1.8	− 1.1	− 8.7	− 4.9	0.013*
Preoperative OKS	4.1 ± 10.9	− 0.6	8.8	1.8	0.085
Postoperative OKS	1.1 ± 7.3	− 2.2	4.4	0.7	0.482
Preoperative EQ-5D	0.2 ± 0.2	− 0.01	0.2	1.9	0.067
Postoperative EQ-5D	0.02 ± 0.1	− 0.1	0.0	− .5	0.572

*P-value < 0.05 indicates the presence of a statistically significant difference

The results of the chi-squared tests for each item of the Swiss Orthopaedics Minimal Dataset Satisfaction Questionnaire in the two cohorts are presented in Table 7.

**Table 7:** The chi-squared test, and the V Cramer statistic, analyzing the outcomes of the Swiss Orthopaedics Minimal Dataset Satisfaction Questionnaire within the two cohorts for each item

	Pearson Chi-squared		Likelihood ratio	
	value	p-value	value	p-value
How satisfied are you right now with the strength of your knee pain when sitting?	1.27	0.88	2.3	0.68
How satisfied are you right now with the strength of your knee pain when you are in bed?	1.58	0.81	2.87	0.58
How satisfied are you right now with the strength of your knee pain when you get out of the bed?	4.05	0.39	4.0	0.4
How satisfied are you currently with the strength of your knee pain when doing light housework?	1.80	0.77	2.79	0.59
How satisfied are you at the moment with the strength of your knee pain when doing recreational activities?	1.95	0.74	2.91	0.57
My expectations regarding pain relief were…	2.44	0.65	3.85	0.42
My expectations regarding the feasibility to do normal everyday activities were…	1.02	0.79	0.97	0.8
My expectations regarding the feasibility to do leisure and sports activities were…	1.88	0.75	2.13	0.71

No intraoperative or postoperative complications were recorded. During the surgical procedure of a 59-year-old male from group 1 a tibial optical robotic array dislocation was reported after burring the implant bed with no further consequence.

## Discussion

The most important finding of this study was that favorable early clinical outcomes were obtained after mUKA in active mid-age and elderly patients presenting with degenerative MMRT and early isolated medial OA or medial SONK. Fast and significant improvements in clinical outcomes and a high level of satisfaction were recorded postoperatively. Moreover, we assessed no difference after the matched pair t-test between the preoperative and postoperative outcomes between the two cohorts, but a significant difference was reached between the results of each cohort postoperatively after mUKA. The patients diagnosed with a d-MMRT and mild OA and similar preoperative symptoms of those knees with severe OA benefit from mUKA with the same favorable results expected from the cohort with primary end-stage bone-on-bone OA. Moreover, the analysis based on the Pearson chi-square tests and the likelihood ratio tests for each item of the Swiss Orthopaedics Minimal Dataset Satisfaction Questionnaire in the two cohorts indicates that there is no significant association between the findings of the two groups.

The age at the time of surgery showed some differences between cohorts. This discrepancy can be partially explained by the distinct populations each cohort represents in terms of the indication for surgery. Another source of difference can be attributed to the non-randomized design of the current study and the inclusion of a limited sample size in Group 1, which may contribute to such disparities.

Arthroscopic pullout repair is still considered the best option for patients who have excellent chondral health and relatively acute meniscal root tears [12, 26–28]. Good potential clinical results presented by MMRT repair tend to diminish if the intervention does not occur within the first weeks after the injury, with cartilage degeneration or subchondral bone abnormalities emerging after months from repair surgery, especially in elderly patients [29–33]. Moreover, varus alignment represents a negative prognostic factor for the success of surgical repair, showing similar clinical results compared to conservative treatment otucomes [34]. Few unambiguous results also have been reported about the postoperative progression of the K–L grade of patients following pullout repair which seems to account for 4–49% of the cases [35–37]. As a matter of fact, limited mid-term evidence evaluating non-active young adult patients with meniscal extrusion and early phase radiographic OA reports good clinical outcomes after MMRT repair but fails to provide strong evidence in such population [14]. Nonetheless, the surgical repair of a MMRT is often associated with a long recovery time from surgery.

Thus, mUKA could represent a reliable option for the treatment of patients with a meniscal extrusion and mild radiographic OA without bone-on-bone contact providing a faster recovery compared to the classical pull-out arthroscopic repair of a degenerative MMRT. Moreover, unwillingness to undergo a higher number of surgical interventions that could result in higher morbidity rates, social, domestic, and health care costs, and psychological sacrifice from patients presenting d-MMRT could represent a new indication of mUKA.

In previously reported studies based on short-, mid-, and long- term follow-up, varus alignment of the affected knee was found to be an independent negative prognostic factor after root repair increasing the risk of medial OA progression and functional deterioration [28, 38, 39]. In our collective, we have found a predominantly varus morphotype with a preoperative mean HKA of 176°, representing a negative outcome predictor for isolated meniscal intervention Thus, the aforementioned assumption and the favorable clinical results within our population could further reinforce the indication for mUKA in treating root tears within this specific demographic. Hiranaka et al. reported the favorable results of their study evaluating the clinical and radiographic outcomes of mUKA for medial compartmental OA following untreated MMRT. The study included a radiographic diagnosis of isolated medial compartmental OA with a K-L grade ranging from 2 to 4. As mentioned above, midterm outcomes of MMRT repair showed that 49% of the knees had radiographic progression of at least 1 grade in the Kellgren-Lawrence scale at a mean follow-up of 4 years and patients over 63.5 years appear to have a lesser risk of osteoarthritis progression [36, 40]. Our study aimed to evaluate patients with fewer degrees of K-L OA regardless of the presence of SONK but still showed encouraging results in terms of PROMs and patient satisfaction.

Only a few studies evaluating the outcomes of mild medial OA of the knee treated with mUKA have been published. Maier et al. reported equal clinical results for partial and full-thickness cartilage loss but with higher revision rates for the first group [41]. Hamilton et al. showed worse results after UKA, with a higher incidence of re-operations in knees with partial thickness cartilage loss in the medial compartment, compared with knees with full thickness loss [42].

Tagliero et al. compared the outcomes of mUKA implanted in a cohort of patients with a meniscal root tear and early OA, and a cohort of patients with advanced primary medial compartment OA [43]. Their results showed that patients with a meniscus root tear had significantly less radiographic arthritis at the time of knee arthroplasty, but similar post-operative clinical outcomes. The study also reported higher functional outcome scores in the meniscus root tear cohort compared to the matched control OA cohort. These findings support our study hypothesis whereby MMRT with meniscal extrusion could be successfully treated with mUKA with good early clinical results independently from the presence of a bone-on-bone OA.

In a meta-analysis of retrospective observational studies, cemented UKA showed similar survival and clinical outcomes in SONK and in medial OA. These findings in cemented UKA could result from the pathologic property of SONK where poor edematous stock present after bone cuts for UKA may affect the survival of uncemented UKA, but have little effect on bone porosity, which affects interdigitation of bone and cement in cemented UKA [44–47]. Wu et al. reported good clinical outcomes for mUKA to treat SONK patients with improved pain and functional status of the affected knee postoperatively, showing mUKA to be a better choice for SONK with only single-compartment involvement compared to TKA. In their study, they confirmed mUKA advantage of minimizing bone stock destruction, decreasing the use of bone cement, and better-preserving knee function in comparison to outcomes following TKA [48]. Although the current study does not primarily investigate SONK lesion, favorable clinical and functional results occur postoperatively for mUKA after unicompartimental SONK patients regardless SONK classification grading.

This study has several limitations. First, the postoperative follow-up period was too short and did not allow for an appropriate evaluation of the long-term clinical outcomes of UKA, especially for failure and revision rates. The favorable result obtained within the first cohort could be exposed to the confounding effect of the exposed younger population in the first cohort which may be fitter to better sustain the mid-term outcomes reported in this study. Nevertheless, the purpose of the current study was to focus on early clinical outcomes just to show that the mUKA in such specific patients’ population offers a fast recovery after surgery not possible with an arthroscopic MMRT repair. Second, this report is a two cohort case series, not a comparative study, and the sample size of the first cohort was small. Thus, the statistical power and validity of the study design regard the role of the second cohort was also small. Third, the validity of the operative indications used in this study is still controversial in the literature and further reports need to confirm the current findings.

Following the favorable improvements in clinical outcomes noted postoperatively in the current study, a novel strand of research aiming to investigate clinical and radiological improvement after mUKA in young and active patients following a d-MMRT diagnosis would be of great interest according to our findings.

As mentioned before, relatively acute meniscal root tears have an indication of arthroscopic pullout repair, conversely, knee arthroplasty is the preferred choice in chronic or degenerative root tears in patients with dominant preexisting osteoarthritis. Thus, a comparative study investigating which is the preferable surgical treatment for subacute onset and which is the cut-off timing demarcating the two different indications should be performed.

Finally, comparative long-term studies to evaluate the clinical results in a population of patients with MMRT and early phase OA treated either with mUKA or with pull-out repair should be performed in order to settle which could be the preferable treatment for such a specific group of patients.

## Conclusions

Favorable clinical outcomes were obtained after mUKA in active mid-age and elderly patients with degenerative MMRT and early isolated medial OA. Patients with mild no bone-on-bone OA but with d-MMRT and MME presented the same or even worse pre-operative symptoms as patients with end-stage medial OA and benefit the same from mUKA.

## Data Availability

The data that support the findings of this study are available on request from the corresponding author.

## References

[1] Ahmed AM, Burke DL, Yu A (1983) In-vitro measurement of static pressure distribution in synovial joints–Part II: retropatellar surface. J Biomech Eng 105:226–236 6632824 10.1115/1.3138410

[2] Fukubayashi T, Kurosawa H (1980) The contact area and pressure distribution pattern of the knee. A study of normal and osteoarthrotic knee joints. Acta Orthop Scand 51:871–879 10.3109/17453678008990887 6894212

[3] Debieux P et al (2021) Medial meniscal extrusion greater than 4 mm reduces medial tibiofemoral compartment contact area: a biomechanical analysis of tibiofemoral contact area and pressures with varying amounts of meniscal extrusion. Knee Surg Sports Traumatol Arthrosc 29:3124–3132 33221933 10.1007/s00167-020-06363-0

[4] Zhan H et al (2023) Radiographic OA, bone marrow lesions, higher body mass index and medial meniscal root tears are significantly associated with medial meniscus extrusion with OA or medial meniscal tears: a systematic review and meta-analysis. Knee Surg Sports Traumatol Arthrosc 31:3420–3433. 10.1007/s00167-023-07418-8 37099153 10.1007/s00167-023-07418-8

[5] Berthiaume M-J et al (2005) Meniscal tear and extrusion are strongly associated with progression of symptomatic knee osteoarthritis as assessed by quantitative magnetic resonance imaging. Ann Rheum Dis 64:556–563 15374855 10.1136/ard.2004.023796 PMC1755443

[6] Gale DR et al (1999) Meniscal subluxation: association with osteoarthritis and joint space narrowing. Osteoarthritis Cartilage 7:526–532 10558850 10.1053/joca.1999.0256

[7] Snoeker BAM, Bakker EWP, Kegel CAT, Lucas C (2013) Risk factors for meniscal tears: a systematic review including meta-analysis. J Orthop Sports Phys Ther 43:352–367 23628788 10.2519/jospt.2013.4295

[8] Sukopp M et al (2021) Influence of menisci on tibiofemoral contact mechanics in human knees: a systematic review. Front Bioeng Biotechnol 9:765596 34926419 10.3389/fbioe.2021.765596 PMC8681859

[9] Furumatsu T et al (2017) Meniscal extrusion progresses shortly after the medial meniscus posterior root tear. Knee Surg Relat Res 29:295–301 29172390 10.5792/ksrr.17.027 PMC5718799

[10] Bhatia S, LaPrade CM, Ellman MB, LaPrade RF (2014) Meniscal root tears: significance, diagnosis, and treatment. Am J Sports Med 42:3016–3030 24623276 10.1177/0363546514524162

[11] Lee JK et al (2020) Repair versus nonrepair of medial meniscus posterior root tear: a systematic review of patients’ selection criteria, including clinical and radiographic outcomes. Medicine (Baltimore) 99:e19499 10.1097/MD.0000000000019499 PMC7478593 32150112

[12] Krivicich LM et al (2022) Comparison of long-term radiographic outcomes and rate and time for conversion to total knee arthroplasty between repair and meniscectomy for medial meniscus posterior root tears: a systematic review and meta-analysis. Am J Sports Med 50:2023–2031 34251898 10.1177/03635465211017514

[13] Wang L, Zhang K, Liu X, Liu Z, Yi Q, Jiang J, Xia Y (2021) The efficacy of meniscus posterior root tears repair: a systematic review and meta-analysis. 10.1177/23094990211003350 10.1177/23094990211003350 33832364

[14] Ro K-H, Kim J-H, Heo J-W, Lee D-H (2020) Clinical and radiological outcomes of meniscal repair versus partial meniscectomy for medial meniscus root tears: a systematic review and meta-analysis. Orthop J Sports Med 8:2325967120962078 33241058 10.1177/2325967120962078 PMC7675875

[15] Krych AJ, Hevesi M, Leland DP, Stuart MJ (2020) Meniscal Root Injuries. J Am Acad Orthop Surg 28:491–499 31693530 10.5435/JAAOS-D-19-00102

[16] Chang PS, Radtke L, Ward P, Brophy RH (2022) Midterm outcomes of posterior medial meniscus root tear repair: a systematic review. 10.1177/0363546521998297 10.1177/0363546521998297 33780278

[17] Kozinn SC, Scott R (1989) Unicondylar knee arthroplasty. J Bone Joint Surg Am 71:145–150 2643607

[18] Pandit H et al (2015) The clinical outcome of minimally invasive Phase 3 Oxford unicompartmental knee arthroplasty: a 15-year follow-up of 1000 UKAs. Bone Jt J 97-B:1493–1500 10.1302/0301-620X.97B11.35634 26530651

[19] Panni AS, Vasso M, Cerciello S, Felici A (2012) Unicompartmental knee replacement provides early clinical and functional improvement stabilizing over time. Knee Surg Sports Traumatol Arthrosc 20:579–585 21748391 10.1007/s00167-011-1613-y

[20] Leppänen S, Niemeläinen M, Huhtala H, Eskelinen A (2021) Mild knee osteoarthritis predicts dissatisfaction after total knee arthroplasty: a prospective study of 186 patients aged 65 years or less with 2-year follow-up. BMC Musculoskelet Disord 22:657 34353317 10.1186/s12891-021-04543-8 PMC8344222

[21] Dawson J, Fitzpatrick R, Murray D, Carr A (1998) Questionnaire on the perceptions of patients about total knee replacement. J Bone Joint Surg Br 80:63–69 9460955 10.1302/0301-620x.80b1.7859

[22] Herdman M et al (2011) Development and preliminary testing of the new five-level version of EQ-5D (EQ-5D-5L). Qual Life Res 20:1727–1736 21479777 10.1007/s11136-011-9903-x PMC3220807

[23] Jentzsch T, Dora C, Müller U, Farshad M (2020) Swiss orthopaedics minimal dataset: first pilot report of reliability and validity. Adv Orthop 2020:e6673175

[24] Matassi F et al (2022) Robotic-assisted unicompartmental knee arthroplasty reduces components’ positioning differences among high- and low-volume surgeons. J Knee Surg 35:1549–1555 33853154 10.1055/s-0041-1727115

[25] Kohn MD, Sassoon AA, Fernando ND (2016) Classifications in brief: Kellgren-Lawrence classification of osteoarthritis. Clin Orthop Relat Res 474:1886 26872913 10.1007/s11999-016-4732-4 PMC4925407

[26] Faucett SC et al (2019) Meniscus root repair vs meniscectomy or nonoperative management to prevent knee osteoarthritis after medial meniscus root tears: clinical and economic effectiveness. Am J Sports Med 47:762–769 29517925 10.1177/0363546518755754

[27] Bernard CD et al (2020) Medial meniscus posterior root tear treatment: a matched cohort comparison of nonoperative management, partial meniscectomy, and repair. Am J Sports Med 48:128–132 31765234 10.1177/0363546519888212

[28] Moon H-K et al (2012) Prognostic factors of arthroscopic pull-out repair for a posterior root tear of the medial meniscus. Am J Sports Med 40:1138–1143 22316547 10.1177/0363546511435622

[29] Moon H-S et al (2020) Early surgical repair of medial meniscus posterior root tear minimizes the progression of meniscal extrusion: 2-year follow-up of clinical and radiographic parameters after arthroscopic transtibial pull-out repair. Am J Sports Med 48:2692–2702 32730732 10.1177/0363546520940715

[30] Sundararajan SR, Ramakanth R, Sethuraman AS, Kannan M, Rajasekaran S (2022) Correlation of factors affecting correction of meniscal extrusion and outcome after medial meniscus root repair. Arch Orthop Trauma Surg 142:823–834 33907865 10.1007/s00402-021-03870-8

[31] Krych AJ et al (2021) Association between transtibial meniscus root repair and rate of meniscal healing and extrusion on postoperative magnetic resonance imaging: a prospective multicenter study. Orthop J Sports Med 9:23259671211023776 34423058 10.1177/23259671211023774 PMC8371730

[32] Kaplan DJ et al (2018) Increased extrusion and ICRS grades at 2-year follow-up following transtibial medial meniscal root repair evaluated by MRI. Knee Surg Sports Traumatol Arthrosc 26:2826–2834 29098324 10.1007/s00167-017-4755-8

[33] Young BL et al (2023) Clinical and radiologic outcomes after meniscal root repair: a case series. J Knee Surg 36:971–976 35901800 10.1055/s-0042-1755421

[34] Comparison between conservative treatment and arthroscopic pull-out repair of the medial meniscus root tear and analysis of prognostic factors for the determination of repair indication | SpringerLink. 10.1007/s00402-015-2269-8 10.1007/s00402-015-2269-8 26142540

[35] Chung KS, Ha JK, Ra HJ, Kim JG (2016) A meta-analysis of clinical and radiographic outcomes of posterior horn medial meniscus root repairs. Knee Surg Sports Traumatol Arthrosc 24:1455–1468 26493550 10.1007/s00167-015-3832-0

[36] Chang PS, Radtke L, Ward P, Brophy RH (2022) Midterm outcomes of posterior medial meniscus root tear repair: a systematic review. Am J Sports Med 50:545–553 33780278 10.1177/0363546521998297

[37] Lee DW, Kim MK, Jang HS, Ha JK, Kim JG (2014) Clinical and radiologic evaluation of arthroscopic medial meniscus root tear refixation: comparison of the modified Mason-Allen stitch and simple stitches. Arthrosc - J Arthrosc Relat Surg 30:1439–1446 10.1016/j.arthro.2014.05.029 25113259

[38] Chung KS, Ha JK, Ra HJ, Kim JG (2021) Preoperative varus alignment and postoperative meniscus extrusion are the main long-term predictive factors of clinical failure of meniscal root repair. Knee Surg Sports Traumatol Arthrosc 29:4122–4130 33730189 10.1007/s00167-020-06405-7

[39] Chung KS, Ha JK, Ra HJ, Kim JG (2016) Prognostic factors in the midterm results of pullout fixation for posterior root tears of the medial meniscus. Arthroscopy 32:1319–1327 26952089 10.1016/j.arthro.2015.12.046

[40] JPM | Free Full-Text | Age and meniscal extrusion are determining factors of osteoarthritis progression after conservative treatments for medial meniscus posterior root tear. https://www.mdpi.com/2075-4426/12/12/2004#B22-jpm-12-02004. 10.3390/jpm12122004 PMC9783096 36556225

[41] Maier MW et al (2015) Unicompartmental knee arthroplasty in patients with full versus partial thickness cartilage loss (PTCL): equal in clinical outcome but with higher reoperation rate for patients with PTCL. Arch Orthop Trauma Surg 135:1169–1175 25940127 10.1007/s00402-015-2236-4

[42] Hamilton TW et al (2017) Unsatisfactory outcomes following unicompartmental knee arthroplasty in patients with partial thickness cartilage loss: a medium-term follow-up. Bone Jt J 99-B:475–482 10.1302/0301-620X.99B4.BJJ-2016-1061.R1 28385936

[43] Tagliero AJ et al (2021) Arthritic progression secondary to meniscus root tear treated with knee arthroplasty demonstrates similar outcomes to primary osteoarthritis: a matched case–control comparison. Knee Surg Sports Traumatol Arthrosc 29:1977–1982 32975627 10.1007/s00167-020-06273-1

[44] Yamamoto T, Bullough PG (2000) Spontaneous osteonecrosis of the knee: the result of subchondral insufficiency fracture*. JBJS 82:858 10.2106/00004623-200006000-00013 10859106

[45] Mears SC, McCarthy EF, Jones LC, Hungerford DS, Mont MA (2009) Characterization and pathological characteristics of spontaneous osteonecrosis of the knee. Iowa Orthop J 29:38–42 19742083 PMC2723690

[46] Ramnath RR, Kattapuram SV (2004) MR appearance of SONK-like subchondral abnormalities in the adult knee: SONK redefined. Skeletal Radiol 33:575–581 15249985 10.1007/s00256-004-0777-7

[47] Graham J, Ries M, Pruitt L (2003) Effect of bone porosity on the mechanical integrity of the bone-cement interface. JBJS 85:1901 10.2106/00004623-200310000-00006 14563796

[48] Ma T, Tu Y, Xue H, Wen T, Mei J (2017) Unicompartmental knee arthroplasty for spontaneous osteonecrosis. J Orthop Surg Hong Kong 25:2309499017690328 28231715 10.1177/2309499017690328

